# Role of Ultrasonography and MRI in Acute Hamstring Strains: Diagnostic and Prognostic Insights

**DOI:** 10.3390/diagnostics15091053

**Published:** 2025-04-22

**Authors:** Yusuke Hirahata, Youichi Yasui, Jun Sasahara, Takahiro Inui, Takumi Nakagawa, Hirotaka Kawano, Wataru Miyamoto

**Affiliations:** 1 Department of Orthopaedic Surgery, Teikyo University School of Medicine, Tokyo 173-8605, Japan; y.hirahata@med.teikyo-u.ac.jp (Y.H.); youichiy41@gmail.com (Y.Y.); figo_sasa@yahoo.co.jp (J.S.); takahiro_inui@med.teikyo-u.ac.jp (T.I.); takumi.nakagawa@gmail.com (T.N.); hkawano-tky@umin.net (H.K.); 2 Institute of Sports Science and Medicine, Teikyo University, Tokyo 192-0395, Japan

**Keywords:** acute hamstring injuries, high-resolution ultrasonography, magnetic resonance imaging, athletes, recovery time, diagnostic accuracy

## Abstract

**Objectives:** Hamstring strain injuries are common in elite athletes and affect return-to-sport timelines. Although magnetic resonance imaging (MRI) is the gold-standard method for assessing injury severity, ultrasonography (US) is a more accessible and cost-effective alternative. This study aimed to evaluate the agreement between US and MRI in the diagnosis of hamstring injuries and their prognostic value in predicting recovery. **Methods:** This retrospective study included elite athletes who sustained acute first-time hamstring strains and underwent both MRI and US within five days after injury. The injuries were classified according to location (muscle belly, musculotendinous junction, or tendon) and severity (modified Peetron’s classification). The agreement between the imaging findings and return-to-sports timelines was analyzed. **Results:** US demonstrated a 70% agreement with MRI in identifying injury locations, showing the highest concordance for muscle belly injuries (90%), followed by musculotendinous junction (80%) injuries, but a lower accuracy for tendon injuries (60%). Recovery times differed significantly by location and severity (*p* < 0.01), with tendon grade 3 injuries requiring the longest recovery (383 days) and muscle belly injuries requiring the shortest recovery (16 days). Musculotendinous junction grade 2, tendon grade 1, and tendon grade 2 injuries had similar recovery durations (57–65 days). **Conclusions:** High-resolution US is a reliable diagnostic tool for muscle belly and musculotendinous junction injuries. However, MRI remains essential for high-grade tendon injuries. US serves as the first-line imaging modality, with MRI reserved for cases that require a detailed prognostic assessment. These findings provide guidance for optimizing imaging strategies for hamstring injury management.

## 1. Introduction

Hamstring injuries, particularly acute strains, are among the most common sports-related injuries, accounting for up to 30% of lower-extremity pathologies in athletes [1,2]. The accurate diagnosis of these injuries is critical, especially for high-grade strains that often lead to prolonged recovery and a delayed return to sports [3,4]. Magnetic resonance imaging (MRI) is widely regarded as the gold-standard method for diagnosing hamstring injuries, owing to its superior ability to assess the extent and severity of the tissue damage [3,5]. However, its high cost, limited accessibility, and delayed scheduling make it less practical for immediate clinical decision making [6,7].

Ultrasonography (US) offers a rapid, cost-effective, and portable alternative to diagnose soft tissue injuries [8,9]. Recent advances in high-resolution US have significantly improved its ability to detect muscle injuries [10]. However, its performance in accurately identifying the injury location and extent compared to that of MRI remains inadequately explored. Moreover, although MRI findings have been associated with predicting recovery timelines [11,12,13], evidence on whether US can reliably support similar prognostic assessments is sparse.

For athletes, quickly and cost-effectively assessing the location and severity of hamstring injuries immediately after their occurrence, along with predicting the recovery timeline for returning to sports, could be highly beneficial. However, previous studies have not comprehensively evaluated the agreement between US and MRI in identifying the location of hamstring injuries or examined the clinical relevance of these findings in relation to recovery time.

This study addressed these gaps by clarifying the agreement between US and MRI for identifying the location and extent of acute hamstring injuries. Additionally, it examined the MRI-based differences in recovery times associated with injury characteristics to provide a comprehensive framework for the diagnostic and prognostic utility of these imaging modalities. The hypotheses of this study were that 1. US has a similar ability to identify the location and extent of acute hamstring injuries as that of MRI and 2. tendon injuries require longer recovery times than those required by muscle and musculotendinous injuries.

## 2. Materials and Methods

### 2.1. Study Design and Ethical Considerations

This retrospective observational study was conducted at Teikyo University Sports Science and Medicine Clinic, a tertiary sports clinic specializing in athletic injuries, between April 2019 and March 2022. The study protocol was approved by our Institutional Review Board (Teikyo University Ethical Review Board for Medical and Health Research Involving Human Subjects; approval number: 19-087-2; approval: 5 February 2025). An opt-out informed consent approach was employed, adhering to the institutional guidelines and ethical standards.

### 2.2. Study Population

Patients with suspected acute hamstring strain, who presented to the clinic during the study period, were retrospectively reviewed. To evaluate the agreement between US and MRI findings, patients who underwent both US and MRI within five days of injury were included. Clinical and imaging data were retrospectively collected from electronic medical records.

Acute hamstring strain was diagnosed by two experienced orthopedic surgeons, following clinical protocols routinely used in the clinic. Patients were assessed for clinical signs, such as swelling, tenderness in the posterior thigh, or pain exacerbated by palpation or movement of the affected limb. The presence of one or more of these findings was considered suggestive of hamstring strain. US and MRI were used to confirm the diagnosis, identify the injured muscles, and evaluate the severity and location of the strain. Details of the US and MRI procedures are described in this manuscript. Treatment was conducted according to standard protocols, including rest, ice, compression, and elevation (RICE), during the acute phase. Patients were advised to keep the affected area at rest and gradually resume activities based on their pain levels. Range-of-motion exercises and weight-bearing activities were permitted once the pain at rest subsided, and patients were instructed to avoid any activities that induced pain. Exercise intensity was gradually increased as tolerated, without causing discomfort. Return to sports was determined in collaboration with the athletes’ trainers and coaches. If there was uncertainty about the timing of return, the attending orthopedic surgeons at the clinic provided a final assessment.

Patients were included in this study if they had undergone both US and MRI with data available for review. Eligible participants were elite athletes who had either competed in national tournaments or were professionals. Furthermore, patients were required to attend follow-up visits to the clinic until they returned to their athletic activities. Additionally, patients with simultaneous muscle or skeletal injuries that could compromise the clinical or imaging evaluations were excluded.

### 2.3. Hamstring Muscle Diagnosis Using US

US was used to diagnose biceps femoris, semimembranosus, and semitendinosus muscle injuries. Examinations were performed during the initial consultation by two orthopedic surgeons with over 10 years of experience in sports orthopedic surgery and routine use of musculoskeletal US in clinical practice. A high-resolution ultrasound machine (Sonimage MX1 Snible Yb, Konica Minolta Japan, Inc., Tokyo, Japan) with a linear probe (L11-3) was employed, which had been introduced to the clinic in 2018.

**Figure 1:**
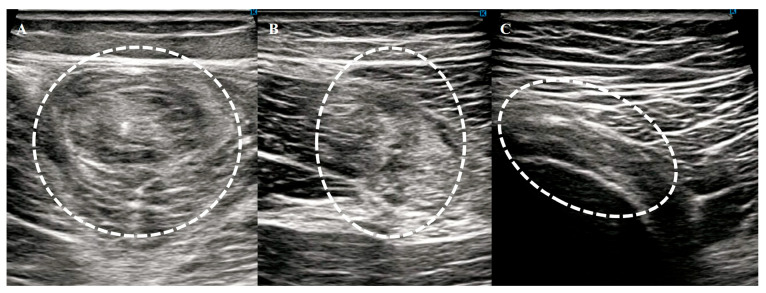
Typical short axis US findings in acute hamstring strains. Muscle (**A**), musculotendinous junction (**B**), and tendon (**C**) injuries. White dotted circles indicate injured areas.

Patients were positioned prone with their hips and knees fully extended, allowing comprehensive scanning of the hamstring muscles from the ischial tuberosity to the knee joint in both the longitudinal and transverse planes. To ensure diagnostic accuracy, US was performed on both the injured and uninjured sides for comparison. An abnormality was diagnosed if one or more of the following findings were observed (Figure 1):

1. Changes in echogenicity or fiber disruption within the muscle.
2. Edema or hemorrhage, defined as areas of increased echogenicity with or without visible fiber disruption in orthogonal planes.
3. Hypoechoic fluid tracking along the fascial layer surrounding the muscle was indicative of intermuscular hematoma.

### 2.4. Hamstring Muscle Diagnosis Using MRI

MRI examinations were conducted using a 0.4-T superconducting unit with a quadrature detection coil (APERTO Lucent, Hitachi, Ltd., Tokyo, Japan). All scans were performed by a radiology technician with over 20 years of experience in musculoskeletal imaging to ensure the consistency and reliability of the imaging process.

The patients were placed in a supine position on the examination table. Both hamstrings were scanned bilaterally from the ischial origin of the muscles to their insertion into the fibula and tibia to comprehensively evaluate the injured and uninjured sides. Imaging was performed in the coronal and axial planes by using short-tau inversion recovery (STIR) sequences. Coronal sequences were obtained with a repetition time (TR)/echo time (TE) of 4660/20 ms, field of view of 340 mm, 224 × 224 matrix, 5 mm section thickness, 0.5 mm gap, and echo-train length of 9. The axial sequences were acquired with a TR/TE of 3800/20 ms, field of view of 340 mm, 224 × 224 matrix, 7 mm section thickness, 1.0 mm gap, and echo-train length of 8.

**Figure 2:**
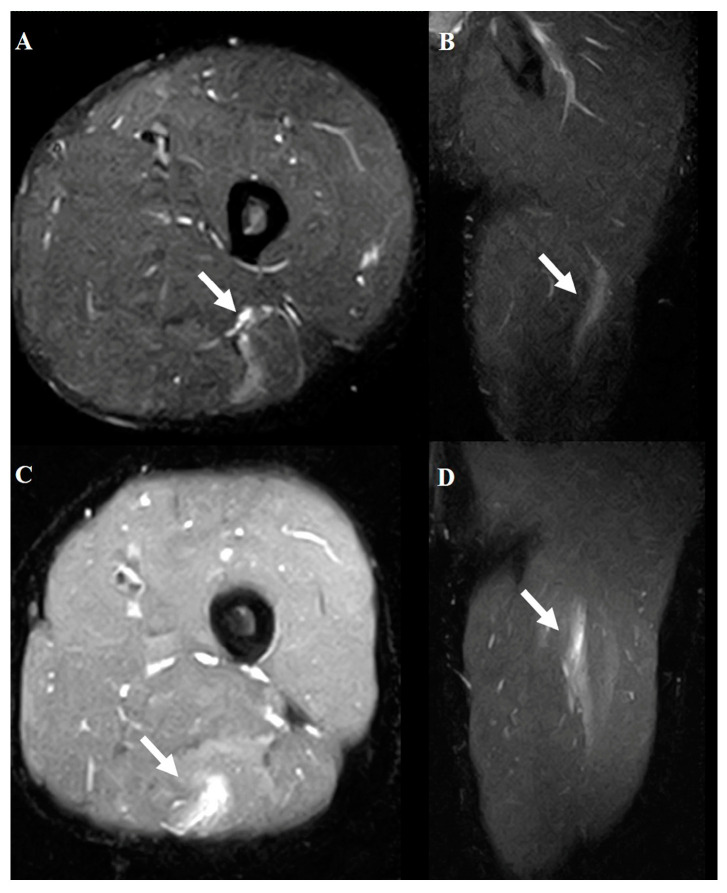
Typical MRI findings in acute hamstring strains. Axial (**A**) and coronal (**B**) views show a grade 1 injury of the biceps femoris muscle. Axial (**C**) and coronal (**D**) views show a grade 2 injury of the semitendinosus muscle. The white arrows indicate the injured areas.

**Figure 3:**
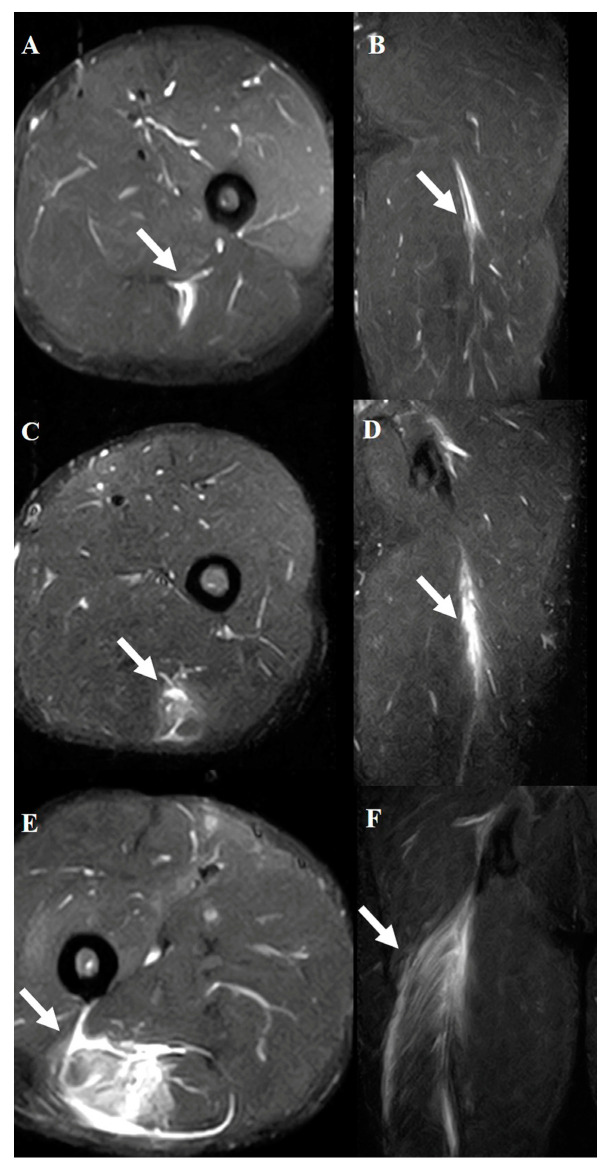
Typical MRI findings in acute hamstring strains. All views show a musculotendinous junction injury of the biceps femoris muscle. Axial (**A**) and coronal (**B**) views of grade 1. Axial (**C**) and coronal (**D**) views of grade 2. Axial (**E**) and coronal (**F**) views of grade 3. The white arrows indicate injured areas.

**Figure 4:**
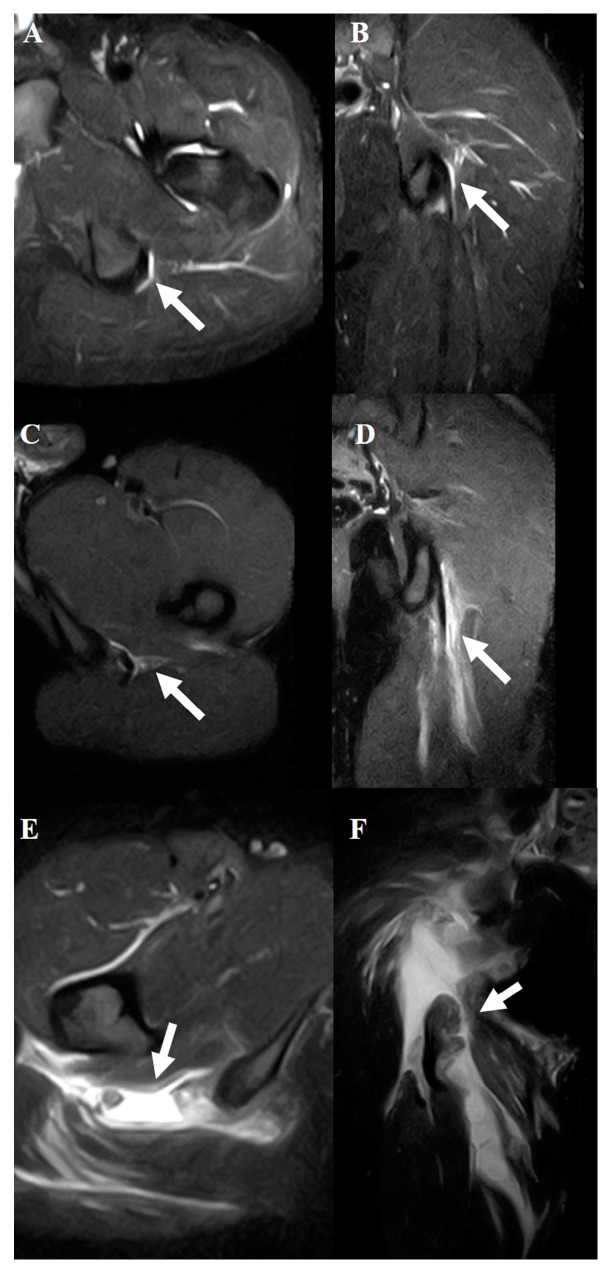
Typical MRI findings in acute hamstring strains. All views show a tendon injury of the semimembranosus muscle. Axial (**A**) and coronal (**B**) views of grade 1. Axial (**C**) and coronal (**D**) views of grade 2. Axial (**E**) and coronal (**F**) views of grade 3. The white arrows indicate injured areas.

The diagnostic criteria were based solely on STIR sequences. These sequences enabled the precise identification of abnormalities, such as edema, hemorrhage, and structural disruptions, in the hamstring muscles. The evaluation focused on identifying the location of the injury (muscle, musculotendinous junction, or tendon) and grading the severity of the injury using the modified Peetron’s classification [14] as follows: grade 0, negative MRI without visible pathology; grade 1, edema without architectural distortion; grade 2, architectural distortion indicating partial tears; and grade 3, total muscle or tendon rupture (Figure 2, Figure 3 and Figure 4).

### 2.5. Evaluation Criteria

The primary objective of this study was to evaluate the agreement between US and MRI findings in diagnosing acute hamstring strain, with a particular focus on identifying injured parts, such as the muscle, musculotendinous junction, or tendon, including avulsion injuries. Metrics such as sensitivity, specificity, positive predictive value (PPV), and negative predictive value (NPV) were calculated to evaluate the diagnostic accuracy of US using MRI as the reference standard.

Additionally, MRI findings were analyzed to explore the distribution of injured muscles and the location and severity of injuries. The recovery times for returning to pre-injury athletic activities were examined across different injury locations and severity grades. The return-to-sport timeline was defined as the interval between the date of injury and the resumption of athletic activities, including training or competitive games, at the pre-injury level. Follow-up consultations and patient interviews provided this information.

### 2.6. Statistical Analysis

Demographic data were described using means and standard deviations for continuous variables and percentages for categorical variables. Differences in the time taken to return to sporting activities were analyzed using one-way analysis of variance (ANOVA) for comparisons among multiple groups. Bonferroni correction was applied for pairwise comparisons to adjust for multiple testing and identify statistically significant differences. STATA (version 16.0; College Station, TX, USA) was used for all analyses. Statistical significance was defined as a two-sided *p*-value < 0.05.

## 3. Results

### 3.1. US’s Diagnostic Accuracy as Part of Agreement Analysis

In a cohort of 109 patients with suspected acute hamstring strains, US identified 66 positive cases, whereas MRI confirmed 71. Using MRI as the reference standard, US demonstrated a sensitivity of 85% (95% confidence interval [CI]: 74.3–91.1%), a specificity of 84% (95% CI: 69.6–92.6%), a positive predictive value of 91% (95% CI: 81.6–95.8%), and a negative predictive value of 73% (95% CI: 59.8–85.1%).

These findings suggest that US can be a practical tool for early diagnosis, particularly in clinical settings where MRI access is limited.

### 3.2. Patient Demographics and Clinical Characteristics

A total of 71 patients met the inclusion criteria and underwent both US and MRI to ensure comprehensive data collection. The detailed demographic characteristics are provided in Table 1, which illustrates the distribution of sports participation, age, and sex within the cohort. Injuries were evenly distributed between the right (48%) and left (52%) sides. Rugby was the most frequently associated sport (49%), followed by sprint running (14%) and baseball (13%). The mean age of the patients was 21 years and 86% were male.

### 3.3. Agreement Between US and MRI and Clinical Relevance of Injury Location

US showed a 70% overall agreement with MRI in identifying the injury locations. The agreement rates varied by muscle group, with the semitendinosus showing the highest agreement (100%), followed by the biceps femoris (82%) and semimembranosus (68%). US identified injuries at the musculotendinous junction in 80% of cases and at the muscle belly in 90% of cases, showing higher detection rates for these locations than for tendon injuries, with an accuracy of 60%.

The MRI findings revealed that the musculotendinous junction was the most commonly injured site (65%), followed by the tendon (21%) and muscle belly (14%). MRI further revealed that the recovery times differed significantly depending on the injury location, with muscle belly injuries recovering in 16 days, musculotendinous junction injuries in 70 days, and tendon injuries in 83 days (*p* < 0.01). Further data supporting these findings are presented in Table 2 and Table 3.

### 3.4. Impact of Injury Severity on Return to Sport Based on MRI Analysis

The injury severity was classified using the modified Peetron’s classification system. Grade 1 injuries accounted for 37%, grade 2 for 32%, and grade 3 for 31% of cases, respectively. The recovery times increased with the injury severity; grade 1 injuries required 35 days, grade 2 injuries required 56 days, and grade 3 injuries required 111 days (*p* < 0.001). These results highlight the necessity of MRI for the assessment of high-grade injuries. The detailed results are presented in Table 4.

## 4. Combined Analysis of Injury Location and Severity

### Summary of US and MRI Utility in Diagnosing Hamstring Injuries

The patients were categorized into nine groups based on the MRI-determined injury location (muscle belly, musculotendinous junction, or tendon) and severity (grades 1, 2, or 3). These findings are presented in Table 5 and Table 6.

- The recovery times showed distinct differences depending on the injury location:
- Tendon grade 3 injuries required the longest recovery time, averaging 383 days, and differed significantly from all other groups (*p* < 0.001).
- Musculotendinous junction grade 3 injuries had a recovery time of 100 days, which was significantly longer than that in most other groups, except for tendon grade 3 injuries (*p* < 0.01).
- The groups with musculotendinous junction grade 2 and tendon grades 1 and 2 injuries showed similar recovery times, ranging from 57 to 65 days.
- Muscle grade 1 injuries had the shortest recovery time, averaging 16 days.

A total of 70 patients underwent conservative treatment and successfully returned to sports. One patient with a tendon grade 3 injury underwent a surgical repair involving the tendon reattachment to the ischial tuberosity using suture anchors. This case highlights the challenges of managing high-grade tendon injuries, as the patient required 383 days to fully recover.

## 5. Discussion

**Figure 5:**
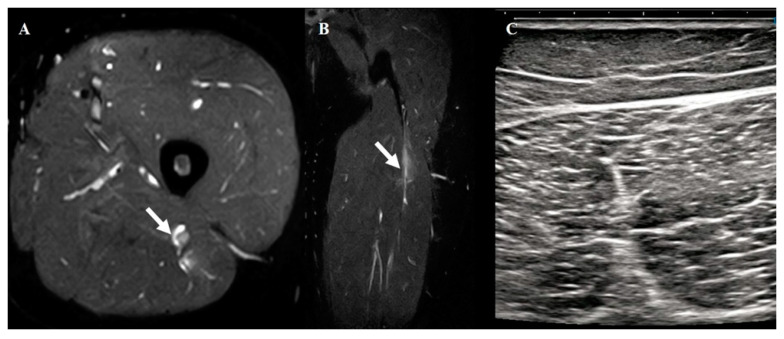
MRI findings of biceps femoris musculotendinous junction injury. Axial (**A**) and coronal (**B**) views of grade 1. White arrows indicate injured areas. (**C**) Short-axis ultrasonography findings in same case did not reveal any injury.

This study highlights the substantial diagnostic utility of high-resolution US for acute hamstring injuries, demonstrating an 85% sensitivity and 70% agreement with MRI in identifying injury locations. US is highly accurate for diagnosing muscle belly (90%) and musculotendinous junction (80%) injuries, which are commonly associated with shorter recovery times. These findings highlight the potential of US as a rapid, accessible, and cost-effective diagnostic tool, particularly in clinical settings where MRI is not readily available. However, US could not detect some grade 1 injuries that had subtle findings on MRI (Figure 5), indicating that US cannot have the same diagnostic value as MRI.

Although the diagnostic accuracy of US is high, its prognostic value remains unclear. MRI findings, including the extent of the muscle tears and the presence of edema, have been strongly correlated with return-to-play timelines [15,16]. This study confirmed that the MRI-identified injury location and severity significantly influenced the recovery time, whereas the role of US in predicting the prognosis requires further investigation. Given its real-time imaging capability, US may serve as an initial assessment tool for triage cases requiring MRI, particularly for moderate injuries involving the muscle belly and musculotendinous junctions.

Few studies have evaluated the radiological diagnostic value of MRI and US for acute hamstring sprains [6,17]. Koulouris and Connell conducted a retrospective study comparing the use of ultrasounds and MRI for the diagnosis of acute hamstring injuries and found that MRI detected proximal hamstring avulsion injuries in 100% of cases, compared to only 58.3% of cases using ultrasound [6]. In contrast, Connell et al. examined 60 patients with acute hamstring strains using sonography and MRI and concluded that both were equally useful for identifying acute hamstring injuries at baseline [17]. In their study, MRI identified abnormalities in 70% (42 of 60 patients) of the cases, whereas sonography revealed abnormalities in 75% (45 of 60 patients). This result showed that 30% of the patients were judged to have a grade 0 injury according to a modification of the Peetron’s classification, showing no signal change on MRI. In the present study, one of the inclusion criteria was patients who underwent MRI after injury and were diagnosed with a proximal hamstring strain by the positive signal change, excluding patients with grade 0 injuries. The subjects selected for sonographic evaluation had signal changes detected by MRI, which were used to investigate how many cases of signal change sonography could depict, which was confirmed by MRI. The two referenced studies [6,17] were conducted in the 2000s using lower-resolution US. Although US technology has advanced since then, no study has yet demonstrated the diagnostic value of high-resolution US in comparison with MRI findings for acute hamstring injuries.

The ability to quickly diagnose the injury location and severity is critical for managing acute hamstring injuries, especially in elite athletes who require precise and timely treatment decisions. US is a valuable first-line diagnostic modality because of its portability and dynamic imaging capabilities. In this study, we performed a combined analysis of the location and severity of muscle injuries. This analysis showed that the most injured part was the musculotendinous junction, followed by the tendons and muscles. Furthermore, the most severe musculotendinous junction and tendon injury, which required a long time to return to sports activity, was a grade 3 injury. Our findings support US’s role in cases involving low- to moderate-grade injuries to the muscle belly or musculotendinous junction, where recovery timelines are predictable and relatively short (16–70 days). These results align with those of previous studies demonstrating the reliability of US in diagnosing soft tissue injuries in the elbow, knee, and ankle [8,9,18,19,20]. However, US has shown a reduced accuracy (60%) in the diagnosis of tendon injuries, which often involve high-grade damage and prolonged recovery periods. In such cases, MRI is indispensable for assessing the extent of the injury and guiding treatment planning. These observations corroborate earlier studies that reported the limitations of US in detecting intratendinous or complex injuries [6,17].

MRI has been extensively used to assess the severity of hamstring injuries and to predict return-to-sport timelines. For example, Ekstrand et al. analyzed 516 conservatively treated hamstring injuries and reported significant differences in the recovery timelines across grades (grade 0, 8 days; grade 1, 17 days; grade 2, 22 days; and grade 3, 73 days; *p* < 0.0001) [21]. Similarly, Cohen et al. found a strong correlation between MRI grades and missed games among professional football players, with grade 3 injuries resulting in the longest absence [22]. In line with these findings, our study confirmed that the MRI-identified injury location and severity significantly influenced the recovery time. Tendon grade 3 injuries required the longest recovery time (383 days), whereas muscle belly injuries required the shortest (16 days). Moreover, moderate injuries involving the musculotendinous junction (grade 2) and tendons (grades 1 and 2) exhibited recovery durations of 57–65 days.

Notably, this study found that musculotendinous junction grade 2, grade 1, and tendon grade 2 injuries had similar recovery times (57–65 days). This suggests that these injuries share common biomechanical and physiological characteristics that influence healing. The clinical implications of this finding warrant further investigation, as they may inform rehabilitation strategies and return-to-play decision making. Future research should explore whether these injury categories respond similarly to treatment and whether additional MRI parameters, such as intramuscular edema patterns or fiber disruption extent, can refine the prognostic accuracy.

Additionally, this study provides novel insights by categorizing the recovery timelines into nine groups based on the injury location and severity. Notably, musculotendinous junction grade 3 and tendon grade 3 injuries required more than 100 days for recovery, corroborating the findings of Askling et al., who associated proximal tendon and muscle–tendon junction injuries with longer recovery times [23]. These detailed classifications offer clinicians a practical framework for setting rehabilitation goals and managing athlete expectations. US, particularly in acute settings, can assist in triaging injuries with shorter recovery times, such as muscle belly and musculotendinous junction injuries.

This study had several limitations. First, the retrospective design and relatively small sample size for certain injury groups, such as muscle grade 3 and tendon grade 3 injuries, limit the generalizability of our findings. Although it would have been ideal to analyze the grade, location, and severity of injuries for each muscle, the limited sample size prevented such detailed analysis. Further studies with larger cohorts are required to validate these findings. Second, the use of a 0.4-T MRI scanner may have restricted the detection of subtle structural abnormalities, although it was sufficient for evaluating the injury location and severity. Third, although the US was performed by experienced sports orthopedic surgeons, inter- and intraobserver reliabilities were not assessed. Future studies should include these evaluations to establish the diagnostic consistency of US. Additionally, as this study focused exclusively on elite athletes, the findings may not be directly applicable to recreational athletes or nonathletic populations. Future investigations should examine whether the diagnostic performances of US and MRI vary across different patient demographics and activity levels.

## 6. Conclusions

This study demonstrated the complementary role of US and MRI in the diagnosis and management of acute hamstring injuries. US has a high diagnostic accuracy for muscle belly and musculotendinous junction injuries, which is combined with its accessibility and cost-effectiveness and supports its use as a first-line imaging tool. However, for high-grade injuries, particularly those involving the tendons, MRI is essential for an accurate diagnosis and treatment planning. This study highlights the importance of optimizing the imaging modality selection. US is effective for diagnosing mild-to-moderate injuries. However, MRI should be prioritized in patients with suspected high-grade tendon involvement, prolonged recovery expectations, or uncertain initial findings. These insights provide a framework for integrating US and MRI into evidence-based clinical pathways, ultimately improving outcomes in athletes with hamstring injuries. Furthermore, advances in US are expected to increase its diagnostic value for acute hamstring injuries. Further studies should explore whether advanced ultrasound techniques (e.g., shear wave elastography) can improve hamstring injury assessments.

## Figures and Tables

**Table 1:** Patient demographics and clinical characteristics.

Variable	Value
Number of patients	71
Sex (% male)	61 (86%)
Age (yr, mean ± SD)	21.0 ± 4.7 (18–48)
Injured side (%)	Right: 48, Left: 52
Sports activity	Rugby: 49, Sprint: 14, Baseball: 13, Soccer: 11, Judo: 8, Long-distance running: 7, American football: 3, Field events: 2, Tennis: 1, Lacrosse: 1
Time to imaging (hr)	US: 57.2 ± 59.3, MRI: 88.9 ± 83.1

**Table 2:** Agreement between US and MRI in diagnosing acute hamstring injuries.

Injury Location	Agreement Rate (%)
Overall Agreement	70
**By Muscle Group**	
Semitendinosus	100
Biceps Femoris	82
Semimembranosus	68
**By Specific Location**	
Musculotendinous Junction	80
Muscle Belly	90
Tendon	60

**Table 3:** MRI findings and return-to-sport timelines.

	Musculotendinous Junction	Tendon	Muscle	*p*-Value
Proportion of Injuries (%)	65	21	14	
Time to Return to Sporting Activity (Days)	70 ± 36	83 ± 90	16 ± 8	<0.01

**Table 4:** Relationship between injury severity (MRI grade) and return-to-sport time.

	Grade 1	Grade 2	Grade 3	*p*-Value
Proportion of Injuries (%)	37	32	31	
The Time to Return to Sporting Activity (Days)	35 ± 28	56 ± 28	111 ± 67	<0.01

**Table 5:** Combined analysis of injury location and severity.

Injured Part	Grade	Number of Patients	Return to Sporting Activity (Days, Mean ± SD)
**Muscle**	1	8	17 ± 8
	2	1	8 ± 0
	3	0	
**Musculotendinous Junction**	1	10	31 ± 13
	2	17	57 ± 21
	3	20	100 ± 27
**Tendon**	1	6	65 ± 39
	2	8	59 ± 37
	3	1	383 ± 0

**Table 6:** Summary of US and MRI utility in diagnosing hamstring injuries.

Group	Muscle Grade 1	Muscle Grade 2	Musculotendinous Grade 1	Musculotendinous Grade 2	Musculotendinous Grade 3	Tendon Grade 1	Tendon Grade 2	Tendon Grade 3
**Muscle Grade 2**	n.s. *							
**Musculotendinous Grade 1**	n.s. *	n.s. *						
**Musculotendinous Grade 2**	<0.05	n.s. *	n.s. *					
**Musculotendinous Grade 3**	<0.001	<0.05	<0.001	<0.001				
**Tendon Grade 1**	<0.05	n.s. *	n.s. *	n.s. *	n.s. *			
**Tendon Grade 2**	<0.05	n.s. *	n.s. *	n.s. *	n.s. *	<0.01		
**Tendon Grade 3**	<0.001	<0.001	<0.001	<0.001	<0.001	<0.001	<0.001	

* n.s. indicates no statistical significance. Statistical values represent comparisons of the recovery timelines across groups.

## Data Availability

The datasets used and/or analyzed in the current study are available from the corresponding author upon reasonable request.

## Abbreviations

MRI	magnetic resonance imaging
US	ultrasonography
